# Resistant Pepper Carrying *N*, *Me1*, and *Me3* have Different Effects on Penetration and Reproduction of Four Major *Meloidogyne* species

**DOI:** 10.21307/jofnem-2019-020

**Published:** 2019-04-23

**Authors:** Abolfazl Hajihassani, William B. Rutter, Xuelin Luo

**Affiliations:** 1 Department of Plant Pathology, University of Georgia, Tifton, GA, 31794; 2 USDA–ARS United States Vegetable Laboratory, Charleston, SC, 294142; 3 University of Georgia, Tifton Campus, Tifton, GA, 31794

**Keywords:** Root-knot nematode, Pepper lines, *Me* gene, Reproduction factor, Gall index, Resistance

## Abstract

Root-knot nematode (*Meloidogyne* spp.) exhibits a substantial problem in pepper production, causing reduction in yield throughout the world. Continued assessment for root-knot resistance is important for developing new resistance cultivars. In this study, the effect of *Me* and *N* genes on the penetration and reproduction of *M. incognita* race 3, *M. arenaria* race 1, *M. javanica,* and *M. haplanaria* was examined under controlled greenhouse conditions using susceptible and resistant pepper lines/cultivars (Mellow Star, Yolo Wonder B, Charleston Belle, HDA-149, HDA-330, PM-217, and PM-687) differing in the presence or absence of resistant genes. The penetration and resistance responses of these pepper lines differed depending on the nematode species. More second-stage juveniles penetrated roots of susceptible control cultivar Mellow Star than roots of resistant cultivars/lines. Although, there was no significant difference in the nematode penetration among resistant lines 1 and 3 days after inoculation (DAI), variability in the penetration of *M. incognita*, *M. javanica*, and *M. haplanaria* was observed 5 DAI. This demonstrates the variability among different nematode resistance genes to invasion by *Meloidogyne* spp. Based on nematode gall index (GI) and reproduction factor (RF), Charleston Belle, HDA-149, PM-217 and PM-687 showed very high resistance (GI < 1 and RF < 0.1) to *M. incognita*, *M. arenaria*, and *M. javanica*. Although, all the *Meloidogyne*-resistant pepper lines evaluated were resistant to *M. javanica* and *M. haplanaria,* the susceptible cultivar Mellow Star was a good host for all nematode species having an RF ranging from 8.1 to 34.7. The *N*, *Me1,* and *Me3* genes controlled resistance to reproduction of all species of *Meloidogyne* examined.

Root-knot nematodes, *Meloidogyne* spp., are one of the most yield-limiting parasites of peppers (*Capsicum annuum* L.) in the USA and worldwide (Sasser and Freckman, 1987; Thies and Fery, 2000). These parasites are widely distributed across the world and are adapted to develop and reproduce on peppers in tropical and subtropical climates. Infection of peppers by *Meloidogyne* spp. can cause changes in the plant physiology, fruit morphology and yield. Three species of *M. incognita* (Kofoid & White) Chitwood, *M. arenaria* (Neal) Chitwood, and *M. javanica* (Treub) Chitwood are particularly significant nematode pests of pepper (Fery et al., 1998; Castagnone-Sereno et al., 2001). *Meloidogyne haplanaria*, Eisenback, Bernard, Starr, Lee & Tomaszewski, a resistant (*Mi* gene)-breaking root-knot species of tomato, has also been reported to infect and reproduce on pepper (Eisenback et al., 2003; Bendezu et al., 2004; Joseph et al., 2016).

Successful management of *Meloidogyne* spp. in pepper includes one or combination of several tactics including rotation with non-host crops, chemical control using fumigant and non-fumigant nematicides, use of resistant cultivars, and other options. Soil fumigation with chemical products is widely used in pepper production in the Southern USA for the control of parasitic nematodes, soilborne pathogens, and weeds. Although some fumigant nematicides are currently available, they do not provide the level of control that was previously observed with methyl bromide and are more challenging to use by most growers (by including fumigant management plans and buffer zones). Additionally, use of non-fumigant nematicides is considered to be a short-term option for the control of root-knot nematodes on peppers grown on plasticulture systems with nematode population levels increasing at the end of the growing season. Since non-fumigant nematicides must be applied season after season, they are prohibitively costly for growers. Also, rotating pepper with non-host crops is not a long-term management option in limiting *Meloidogyne* spp. damage because of wide host range of the nematode (Trudgill and Blok, 2001). For these reasons, research efforts have been directed toward the development of sustainable and eco-friendly nematode management approaches.

In this context, the use of resistant cultivars with acceptable growth and yield characteristics appears to be an effective strategy to manage plant-parasitic nematodes, in particular root-knot nematodes (Hendy et al., 1985; Thies and Fery, 2000; Pegard et al., 2005). Resistance to root-knot nematode infection is established as an inhibition or decrease of nematode penetration and/or reproduction (Trudgill, 1991; Williamson and Kumar, 2006) and is characterized by a localized hypersensitive reaction in host plants (Pegard et al., 2005). The development of successful *Meloidogyne*-resistant pepper breeding programs is dependent on the characterization of new resistant pepper lines. Multiple dominant resistance genes effective against several species of root-knot nematodes have been discovered in the relative and wild species of peppers (Hendy et al., 1985). These genes are denoted as *N*, *Me1*, *Me2*, *Me3* (*=Me7*), *Me4*, *Me5*, *Me6*, *Mech1*, and *Mech2* (Hendy et al., 1985; Djian-Caporalino et al., 2001, 2007). Three of these genes (*N*, *Me1*, and *Me3*) are broadly effective against the three most widespread tropical root-knot nematode species (*M. incognita*, *M. javanica*, and *M. arenaria*). Pepper cultivars/lines carrying different resistant genes differ in their ability to withstand invasion and reproduction of different species or populations of root-knot nematodes (Bleve-Zaccheo et al., 1998; Pegard et al., 2005).

In the USA, investigation of resistance to root-knot nematodes in peppers has been restricted primarily to pepper lines carrying the *N* gene (Fery et al., 1998; Thies and Fery, 2000; Thies, 2011). Four isogenic lines (HDA-149, HDA-330, PM-217, and PM-687) were developed in France to be incorporated into pepper breeding programs. These lines carry additional resistance genes against *Meloidogyne* spp. and have been extensively evaluated against the *M. incognita* group species (MIG) (Hendy et al., 1985, Djian-Caporalino et al., 2001, 2007). However, the levels of resistance in these lines have not been assessed against populations of *M. arenaria* race 1 and *M. javanica* from the Southern USA, and to our knowledge these resistance genes have never been assessed for their efficacy against *M. haplanaria*. *Meloidogyne haplanaria* is a recently described species from the southern USA that is known to overcome the widely used *Mi* gene in tomato, but has not been evaluated against resistance genes from other solonacious crops (Eisenback et al., 2003, Bendezu et al., 2004, Joseph et al., 2016). Due to the well documented hyper variability of the MIG species, and their ability to break resistance in pepper lines carrying the *Me3* and *N* genes (Castagnone-Sereno et al., 2001; Thies, 2011, Bucki et al., 2017), it is imperative to continue assessing the existing resistant pepper lines against different populations and species of *Meloidogyne*. The aim of this study was to examine the penetration and reproduction responses of HDA-149, HDA-330, PM-217, and PM-687 to *M. incognita* race 3, *M. arenaria* race 1, *M. javanica*, and *M. haplanaria*, and compared their responses to the *Meloidogyne*-resistant cultivars, Yolo Wonder B and Charleston Belle (Fery et al., 1998).

## Materials and methods

### Nematode inoculum

Pure cultures of *M. incognita* race 3 and *M. arenaria* race 1 were obtained from P. Timper and R. Davis (USDA–ARS, Tifton, GA), culture of *M. javanica* was obtained from J. Noe (University of Georgia, Athens), and culture of *M. haplanaria* was obtained from T. Mengistu (Formerly at University of Florida, Gainesville). These nematode isolates were multiplied separately on coleus (*Plectranthus* sp.) plant in the greenhouse for 15 weeks. Second-stage juveniles (J2) of each *Meloidogyne* spp. isolate were recovered from the infected coleus roots by incubating chopped roots in a mist chamber for five days.

### Pepper cultivars/lines

The pepper cultivars/lines used were Mellow Star (susceptible control pepper; Johnny’s selected seeds, Maine), Yolo Wonder B, Charleston Belle, HDA-149, HDA-330, PM-217, and PM-687. Yolo Wonder B is resistant to *M. arenaria* and its resistance is conferred by the *Me5* gene. Charleston Belle is resistant to *M. incognita, M. arenaria* races 1 and 2, and *M. javanica* and its resistance is conditioned by the *N* gene (Fery et al., 1998; Thies and Fery, 2000). HDA-149 and HDA-330 are double haploid lines that harbored the *Me3* and *Me1* genes, respectively and conferred resistance to *M. incognita, M. arenaria*, and *M. javanica*. PM-217 and PM-687 have the *Me1* and *Me3* genes, respectively for resistance to *M. incognita, M. arenaria, M. javanica* (Hendy et al., 1985; Berthou et al., 2003).

### Penetration study

Pepper seeds were planted in 128-cell plug trays (Speedling Incorporated, Ruskin, FL) filled with Miracle-Gro Moisture Control potting mix (The Scotts Miracle-Gro Company, Marysville, OH) two to three weeks before nematode inoculation. Pepper seedlings, at two true leaf stage and approximately 6 cm tall, were transplanted into Deepot D40L cell containers (6.9-cm-dia. × 25.4-cm deep, vol. 410 mL; Stuewe & Sons, Inc., Tangent, Oregon) containing pasteurized field soil: washed sand (2:1 v/v). Before transplanting, potted soils were watered and seedlings were transplanted individually in cell containers, and then inoculated with 300 J2 in 1 ml water pipetted into two holes (3 cm deep) made in the soil around the plant base. The plants were arranged in a completely randomized design with four replicates on support trays (Stuewe & Sons, Inc., Tangent, Oregon) in a greenhouse at 25±3 ° C. Enough plants were inoculated with *M. incognita* race 3, *M. arenaria* race 1, *M. javanica*, and *M. haplanaria* to allow destructive sampling of three plants every other day up to five days. The pots were watered lightly each day. On each sampling day, three seedlings were randomly taken and harvested to recover the root system. The root systems were washed gently with tap water to remove soil, soaked in 1.5% NaOCL (wt/vol) for 3 min and a final rinse of tap water. The nematode J2 in intact roots were stained by boiling for 30 sec in red food color (Thies et al., 2002). After staining, the roots were rinsed with tap water, destained in lactophenol for 48 hr (Hajihassani et al., 2017). Visualization of nematode J2 in root tissues was done by pressing each root system between two glass slides and examination with a stereomicroscope at ×20 to ×90 magnification. Nematode penetration was assessed by the enumeration of the J2 stained inside the roots. This experiment was repeated once.

### Reproduction study

Pepper seedlings were transplanted into the Deepot D40L cell containers filled with pasteurized field soil: washed sand (2:1 v/v). At transplanting, 1,000 J2 in 1 ml water were pipetted into two holes (2-3-cm deep) made in the soil around the plant base. The plants were arranged in a completely randomized design with five replicates on support trays in the greenhouse. Plants were watered once a day with equal amounts of water, fertilized once during the experiment with 10-g Osmocote smart-release fertilizer (15-9-12, The Scotts, Marysville, OH), grown at 28 ± 3°C for eight weeks, at which time root systems were harvested, washed gently with running water, air dried briefly on paper towels, and weighed. The root systems were stained as described previously and then rated for nematode reproduction with a gall index (GI) using a 0 to 5 scale as follow: 0 = no gall; 1 = 1 to 2 galls on root system, 2 = 3 to 10 galls, 3 = 11 to 30 galls, 4 = 31 to 100 galls, and 5 =>100 galls (Taylor and Sasser, 1978). Eggs were extracted from root systems separately using the NaOCl method (Hussey and Barker, 1973) and counted under an inverted microscope. Nematode reproduction was also measured by calculating the reproduction factor (RF: final number of nematodes/initial number inoculated). The GI and RF are important measures of resistance of a plant species to *Meloidogyne* spp. (Sasser et al., 1984). These measurements were selected as the primary parameters in determining resistance/susceptibility in pepper cultivars/lines against *Meloidogyne* spp. The experiment was repeated once.

### Analysis of data

A two-way analysis of variance (ANOVA) using PROC Mixed within SAS (v. 9.2, SAS Institute, Cary, NC) was performed on data obtained in the penetration and reproduction studies. Since no significant differences in the fresh root weight, egg counts, GI, and RF were observed between two trials in both penetration (*p* > *F* = 0.08) and reproduction (*p* > *F* = 0.1) studies, data were grouped for statistical analysis. Means were separated with Tukey’s adjustment for multiple comparisons test. The confidence interval for statistical significance was 95%.

## Results

### Penetration study

Nematode penetration as evident from enumeration of the J2 inside the roots was affected by pepper cultivar/lines among all *Meloidogyne* spp. The nematodes penetrated roots of all susceptible and resistant peppers, but a significant effect of plant genotype (*p* < 0.0001), DAI (*p* < 0.0001) and line/cultivar × DAI interaction (*p* < 0.0001) was observed.

By 1 DAI, the number of J2 in the roots of susceptible control cultivar Mellow Star and Yolo Wonder B did not differ for *M. incognita* and *M. arenaria* but differed for *M. javanica* and *M. haplanaria*. A significantly greater number of *M. incognita* and *M. arenaria* J2 entered roots of the susceptible cultivars Mellow Star and Yolo Wonder B compared to the resistant lines Charleston Belle, HDA-149, HDA-330, PM-217, and PM-687. For *M. javanica* and *M. haplanaria*, however, greater numbers of J2 were present in roots of Mellow Star than in roots of other cultivars/lines. No significant difference between numbers of *Meloidogyne* J2 was found among Charleston Belle, HDA-149, HDA-330, PM-217, and PM-687 (Table 1). Similar results in the nematode penetration, except observing a significant difference in the number of *M. incognita* J2 between Mellow Star and Yolo Wonder B, were found at 3 DAI (Table 1). At 5 DAI, although, fewer J2 were present in roots of HDA-149 than in Charleston Belle, HDA-330, PM-217, and PM-687, no significant difference in *M. arenaria* penetration was observed among Charleston Belle, HDA-149, HDA-330, PM-217, and PM-687 (Table 1). At 5 DAI, significantly more J2 of *M. javanica* and *M. haplanaria* penetrated Mellow Star than other cultivars/lines. Also, the number of *M. javanica* J2 in roots of Charleston Belle was numerically lowest, but no significant difference in the nematode penetration was found between this cultivar with other lines of HDA-149, HDA-330, and PM-687.

**Table 1:** Number of *Meloidogyne incognita* race 3, *M. arenaria* race 1, *M. javanica,* and *M. haplanaria* in roots of susceptible and resistant pepper lines at 1, 3, and 5 days after inoculation.

	Days after nematode inoculation		
Cultivar/line (resistant gene)	1	3	5
*M. incognita race 3*			
Mellow Star^X^	11.0±1.24 a	21.8±2.47 a	39.0±4.50 a
Yolo Wonder B (*Me5*)^Y^	8.1±1.16 a	15.0±1.74 b	38.1±3.9 a
Charleston Belle (N)^Y^	0.6±0.30 b	3.8±0.72 c	7.5±0.90 bc
HDA-149 (*Me3*)	0.3±0.30 b	3.0±0.60 c	5.1±0.74 c
HDA-330 (*Me1*)	0.8±0.45 b	7.3±0.83 c	12.0±1.16 b
PM-217 (*Me1,Me2*)	0.5±0.30 b	6.3±1.32 c	10.6±2.60 b
PM-687 (*Me3,Me4*)	0.5±0.30 b	3.8±1.20 c	6.8±1.30 bc
*M. arenaria race 1*			
Mellow Star	10.1±1.45 a	17.1±2.93 a	35.2±6.0 a
Yolo Wonder B	7.8±1.85 a	12.6±1.74 a	37.0±5.8 a
Charleston Belle	0.1±0.16 b	2.3±0.77 b	5.1±1.4 b
HDA-149	0.1±0.16 b	3.0±0.77 b	10.6±1.1 b
HDA-330	0.6±0.30 b	5.0±0.78 b	10.3±0.8 b
PM-217	0.3±0.30 b	5.5±0.86 b	9.5±1.1 b
PM-687	0.3±0.30 b	4.6±1.67 b	6.6±1.2 b
*M. javanica*			
Mellow Star	9.6±0.9 a	18.7±2.3 a	30.7±4.5 a
Yolo Wonder B	0.5±0.06 b	3.8±1.0 b	9.8±1.2 b
Charleston Belle	0.1±0.02 b	2.1±0.7 b	4.6±1.3 c
HDA-149	0.5±0.03 b	4.8±1.0 b	9.0±2.6 bc
HDA-330	0.6±0.1 b	3.6±1.3 b	5.8±1.1 bc
PM-217	0.3±0.02 b	4.8±1.4 b	9.3±2.3 b
PM-687	0.3±0.02 b	1.8±0.72 b	6.3±0.94 bc
*M. haplanaria*			
Mellow Star	7.2±1.04 a	16.6±2.03 a	21.1±2.1 a
Yolo Wonder B	0.6±0.18 b	4.5±0.8 b	10.1±1.1 bc
Charleston Belle	0.2±0.04 b	2.5±0.8 b	4.5±1.0 d
HDA-149	0.3±0.16 b	3.9±0.7 b	8.3±1.2 bc
HDA-330	0.3±0.06 b	5.6±1.6 b	9.6±2.1 bc
PM-217	0.3±0.06 b	3.6±0.9 b	10.8±1.1 b
PM-687	0.6±0.03 b	4.3±1.3 b	7.1±1.7 cd

**Notes:** ^X^susceptible control; ^Y^resistant control. Each plant was inoculated with 300 second-stage juveniles of each nematode species at transplanting. For each nematode species, each value represents the mean ± standard error (*n* = 6) of each treatment. Means followed by the same letter within columns are not significantly different (*p* = 0.05) based on Tukey’s test.

### Reproduction study

There was no significant difference in fresh root weight among the pepper lines infected with all *Meloidogyne* spp. In contrast, significant differences were observed for GI (*p* < 0.0001), egg counts/g fresh root (*p* < 0.0001), and RF (*p* < 0.0001) (Table 2).

**Table 2:** Root fresh weight, gall index, numbers of eggs per gram fresh root mass, and reproduction factor of *Meloidogyne incognita* race 3, *M. arenaria* race 1, *M. javanica,* and *M. haplanaria* on pepper cultivar/lines in two greenhouse trials.

Cultivar/line	Root weight	Gall index^Y^	Eggs/g root	Reproduction factor^Z^
M. incognita race 3				
Mellow Star^X^	11.6±1.2 a	3.7±0.6 a	2,966.5±769.1 b	33.9±7.37 b
Yolo Wonder B^W^	11.2±1.4 a	4.0±0.5 a	4,387.4±1,037.6 a	48.1±9.87 a
Charleston Belle^W^	10.1±1.7 a	0.5±0.3 b	73.2±12.3 c	0.6±0.09 c
HDA-149	10.3±1.5 a	0.3±0.3 b	6.5±2.0 c	0.1±0.01 c
HDA-330	11.4±0.9 a	0.7 ±0.2 b	88.4±24.0 c	0.9±0.27 c
PM-217	11.3±1.5 a	0.2±0.3 c	6.7±1.7 c	0.1±0.02 c
PM-687	11.7±0.7 a	0.3±0.3 b	24.4±5.1 c	0.3±0.06 c
*M. arenaria race 1*				
Mellow Star	11.1±1.4 a	3.6±0.7 a	3,205.3±718.1 a	34.7±6.07 a
Yolo Wonder B	10.0±1.5 a	3.1±0.5 a	3,163.4±904.2 a	30.2±9.88 a
Charleston Belle	11.1±1.2 a	0.4±0.4 b	10.6±0.8 b	0.05±0.01 b
HDA-149	10.8±1.7 a	0.4±0.3 b	6.5±1.6 b	0.07±0.02 b
HDA-330	10.1±1.3 a	0.7±0.3 b	32.8±9.3 b	0.31±0.07 b
PM-217	10.7±1.6 a	0.4±0.4 b	8.4±1.7 b	0.08±0.02 b
PM-687	11.5±1.1 a	0.5±0.3 b	3.5±0.9 b	0.04±0.01 b
*M. javanica*				
Mellow Star	11.7±1.3 a	3.7±0.8 a	2,059.5±454.0 a	23.5±4.46 a
Yolo Wonder B	9.4±1.6 a	0.9±0.4 b	5.0± 1.4 b	0.04±0.01 b
Charleston Belle	10.4±1.7 a	0.4±0.4 b	1.9±1.1 b	0.01±0.01 b
HDA-149	9.5±1.5 a	0.4±0.3 b	10.1±3.6 b	0.09±0.02 b
HDA-330	10.3±1.8 a	0.5±0.4 b	8.7±5.6 b	0.06±0.03 b
PM-217	10.1±1.5 a	0.4±0.3 b	24.6±8.9 b	0.24±0.01 b
PM-687	10.1±1.7 a	0.7±0.3 b	1.7±0.7 b	0.01±0.00 b
*M. haplanaria*				
Mellow Star	12.4±1.4 a	2.7±1.25 a	655.0±57.0 a	8.1±1.25 a
Yolo Wonder B	10.3±1.1 a	0.3±0.02 b	5.4±2.4 b	0.06±0.02 b
Charleston Belle	9.3±1.3 a	0.4±0.06 b	20.2±8.9 b	0.17±0.06 b
HDA-149	10.7±1.1 a	0.4±0.04 b	12.7±4.9 b	0.13±0.04 b
HDA-330	10.5±1.1 a	0.7±0.07 b	25.9±7.3 b	0.27±0.07 b
PM-217	10.5±1.1 a	0.4±0.01 b	3.5±1.5 b	0.03±0.01 b
PM-687	12.0±1.2 a	0.6±0.04 b	13.3±2.9 b	0.15±0.03 b

Notes: ^X^susceptible control; ^W^resistant control; Each plant was inoculated with 1,000 second-stage juveniles of each nematode species at transplanting; ^Y^ rated on a scale of 0 to 5: 0 = no gall; 1 = 1 to 2 galls on root system, 2 = 3 to 10 galls, 3 = 11 to 30 galls, 4 = 31 to 100 galls, and 5 ⩾100 galls; ^Z^ Reproduction factor = final number of nematodes/initial number inoculated; Each value represents the mean ± standard error (*n* = 10) of each treatment. Means followed by the same letter within columns are not significantly different (*p* = 0.05) based on Tukey’s test.

While the roots of PM-217 showed lowest GI (GI = 0.2) caused by *M. incognita*, no significant difference for egg counts and RF was observed among the resistant peppers Charleston Belle, HDA-149, HDA-330, PM-217, and PM-687 (Table 2). All isogenic lines had no visible root galling in contrast to Mellow Star and Yolo Wonder B which exhibited high root galling (GI = 3.7 and 4.0, respectively). The number of *M. incognita* eggs produced per gram of root mass was greatest in Mellow Star and Yolo Wonder B compared to all cultivars/lines examined. The RF value of *M. incognita* for Charleston Belle, HDA-149, HDA-330, PM-217, and PM-687 were 0.6, 0.1, 0.9, 0.1, and 0.3, respectively (Table 2). All isogenic lines, except Yolo Wonder B and HDA-330, were highly resistant to *M. incognita* (Table 3). *Meloidogyne arenaria* produced significantly fewer galls and eggs in the roots of Charleston Belle, HDA-149, HDA-330, PM-217, and PM-687 than in Mellow Star and Yolo Wonder B which reproduced aggressively, having an RF of 34.7 and 30.2, respectively (Table 2). Among isogenic lines examined, only Yolo Wonder B was found highly susceptible to *M. arenaria* (Table 3). *Meloidogyne javanica* was highly virulent on Mellow Star producing over 2,000 egg per g root. This cultivar had the highest GI (3.7) and RF value (23.5) as compared to other cultivars/lines that had a RF value < 0.1. No significant difference for the RF was observed among Charleston Belle and four isogenic lines. The RF value of *M. javanica* was higher (RF = 23.5) in Mellow Star than in other pepper cultivars/lines, with the RF values ranging from 0.01 to 0.24 (Table 2). *Meloidogyne haplanaria* reproduced (GI = 2.7, RF = 8.1) well on Mellow Star only, but it was not able to reproduce on the other pepper lines (Table 2). All isogenic lines examined were highly resistant to both *M. javanica* and *M. haplanaria* (Table 3).

**Table 3:** Resistance and susceptibility ratings of pepper plants to species of root-knot nematode (*Meloidogyne* spp.) based on greenhouse experiments.

Cultivar/Line	Nematode resistance genes	*M. incognita* race 3	*M. arenaria* race 1	*M. javanica*	*M. haplanaria*
Mellow Star	None	HS	HS	HS	S
Yolo Wonder B	*Me5*	HS	HS	HR	HR
Charleston Belle	*N*	HR	HR	HR	HR
HDA-149	*Me3* (*=Me7*)	HR	HR	HR	HR
HDA-330	*Me1*	R	HR	HR	HR
PM-217	*Me1, Me2*	HR	HR	HR	HR
PM-687	*Me3* (*=Me7*), *Me4*	HR	HR	HR	HR

**Notes:** Root gall index (GI) and reproduction factor (RF: final number of nematodes/initial number inoculated) were used to assess resistance/susceptibly ratings as follows: HR: highly resistant (GI < 1 and RF < 0.1), R: resistant (GI < 1 and RF ⩽1), S: susceptible (GI ≥ 2 and RF = 1 to 10), and HR: highly susceptible (GI ⩾ 3 and RF ⩾ 10).

## Discussion

The first purpose of this research was to examine the variability in penetration of four species of root-knot nematodes in pepper. We found that *M. incognita* race 3, *M. arenaria* race 1, *M. javanica*, and *M. haplanaria* were capable of entering the root systems of susceptible and resistance cultivar/lines, but the penetration rates were considerably reduced in the resistant cultivars/lines at 1, 3 and 5 DAI. These results are consistent with the results obtained by Pegard et al. (2005) and Bleve-Zaccheo et al. (1998). In our study, between 13 and 30% more *M. incognita* J2s were present in roots of susceptible cultivars Mellow Star and Yolo Wonder B than in roots of all resistant lines Charleston Belle, HDA-149, HDA-330, PM-217, and PM-687 at 5 DAI. There were also significant differences between the resistant lines. Fewer *M. incognita* J2 penetrated the root system of Charleston Belle, HDA-149, and PM-687, than in HDA-330 and PM-217 at 5 DAI (Table 1). This is consistent with Bleve-Zaccheo et al. (1998) which reported that fewer *M. incognita* J2 were present in root of the line HDA-149 (carrying *Me3*) than in HDA-330 (carrying *Me1*) at 4 DAI and which reported that lines carrying *Me1* exhibit a delayed hypersensitive response compared to those carrying *Me3.* Interestingly, we did not see these same differences in the penetration of the other three *Meloidogyne* species tested against these same resistant lines.

This could be the result of other resistance loci outside of the well characterized *Me* genes which may specifically contribute to resistance against these other *Meloidogyne* species. We found that 17 to 29% greater J2 of *M. arenaria* penetrated Mellow Star and Yolo Wonder B root systems than in Charleston Belle, HDA-149, HDA-330, PM-217, and PM-687, respectively at 5 DAI. The numbers of *M. arenaria* penetrated the pepper roots were consistent among all resistant cultivars/lines examined, suggesting that the nematode infection did not affect plant defense response. The numbers of *M. javanica* and *M. haplanaria* J2 present in the root of Mellow Star were greater than the roots of all other cultivars/lines examined. Fewer *M. javanica* J2 penetrated the root system of Charleston Belle, HDA-149, HDA-330, and PM-687, 5 DAI than in Yolo Wonder B and PM-217. The total number of *M. haplanaria* J2 in roots of Charleston Belle 5 DAI was 42 to 63% fewer than in Yolo Wonder B, HDA-149, HDA-330, PM-217, and PM-687, respectively. Our results demonstrate that J2 penetration on the plant vary with species of root-knot nematode and pepper cultivars/lines carrying resistance genes.

Resistance to root-knot nematode infection is established as an inhibition or reduction of nematode reproduction (Trudgill, 1991) or prevention of feeding site establishment (Williamson and Kumar, 2006) in host plants. In the present study, resistance responses of the pepper cultivars/lines differed depending on *Meloidogyne* spp. This variability in resistance to the nematode species was found based on the GI and RF among pepper lines tested. Resistance to *Meloidogyne* spp. in pepper is conferred by several single dominant genes. Some of these genes induce resistance to only one nematode species, while others are effective to a wide range of *Meloidogyne* spp. (Djian-Caporalino et al., 1999). *Meloidogyne incognita* race 3 was highly pathogenic on Mellow Star and Yolo Wonder B producing the GI ⩾ 3 and RF ⩾ 10, respectively. Other lines including Charleston Belle, HDA-149, PM-217, and PM-687 showed high resistance to *M. incognita*. The low RF (Table 3) of *M. incognita* in the resistant pepper lines is thought to be related to the presence of phenolic compounds and chlorogenic acid which reduce nematode penetration and affect adversely nematode development and reproduction in pepper roots (Bleve-Zaccheo et al., 1998; Pegard et al., 2005).

The isogenic line HDA-330 exhibited a GI < 1 and RF ⩽1 in response to *M. incognita*, and was found to be resistant to the nematode. It has been reported that the resistance mediated by the *Me1* gene to *M. incognita* was more durable than that mediated by *Me3* or the *N* gene (Sánchez-Solana et al., 2016; Bucki et al., 2017). Our greenhouse data showed that *M. incognita* slightly reproduced (RF = 0.9) on HDA-330 (*Me1*), though it was not statistically greater than the other resistant lines. Populations of *M. incognita* have been reported to break resistance in pepper lines carrying the *Me3* and *N* gene (Castagnone-Sereno et al., 2001; Piedra Buena et al., 2005; Thies, 2011).

The *M. arenaria*-resistant pepper lines Charleston Belle, HDA-149, HDA-330, PM-217, and PM-687 exhibited high resistance to *M. arenaria* race 1. Mellow Star and Yolo Wonder B, however, supported the nematode reproduction and were found highly susceptible (GI >3 and RF > 30). This is consistent with previous studies which demonstrated that peppers carrying *N, Me1,* and *M3* genes are resistant to populations of *M. arenaria* (Thies and Fery, 2000, 2001; Djian-Caporalino et al., 2007). While Mellow Star was highly susceptible to *M. javanica*, the other six pepper cultivar/lines tested were highly resistant to the nematode, which is consistent with the finding that the *Me5* resistance gene, carried by Yolo Wonder, is specific against *M. javanica* (Hendy et al., 1985).

To our knowledge this was the first time *M. haplanaria* has been tested against any root-knot nematode resistant pepper lines. Interestingly, we found that all the resistant pepper lines, including Yolo Wonder B (*Me5*) were significantly more resistant to *M. haplanaria* compared to our susceptible control Mellow Star (GI = 2.7 2 and RF = 8.1). *Meloidogyne haplanaria* is able to overcome the resistance in tomato mediated by the *Mi* gene (Bendezu et al., 2004). The lack of ability of *M. haplanaria* to damage commercial resistant cultivars of peppers is important for their use in rotation with other vegetable crops for managing this nematode pest in infested fields in the southern USA.

In summary, fewer root-knot nematode J2 penetrated the roots of the resistant peppers than susceptible peppers. This study showed a correlation between the fullness of resistance and lines specificity during the examination of pepper lines carrying different root-knot nematode resistance genes. Our data supports previously observed virulence/avirulence trends for *M. incognita*, *M. javanica*, and *M. arenaria* against pepper lines carrying the *N*, *Me1*, *Me3*(=*Me7*), and *Me5* resistance genes, and has provided evidence that all four of these genes are also likely to be effective against *M. haplanaria*. Identification of the genes and molecular pathways involved in pepper responses and development of genetic markers for resistance will facilitate the breeding programs for the development of peppers with broad and durable *Meloidogyne*-resistance.
